# Broadband-NIRS System Identifies Epileptic Focus in a Child with Focal Cortical Dysplasia—A Case Study

**DOI:** 10.3390/metabo12030260

**Published:** 2022-03-17

**Authors:** Aikaterini Vezyroglou, Peter Hebden, Isabel De Roever, Rachel Thornton, Subhabrata Mitra, Alan Worley, Mariana Alves, Emma Dean, Judith Helen Cross, Ilias Tachtsidis

**Affiliations:** 1 Developmental Neurosciences, UCL Great Ormond Street Institute of Child Health, London WC1N 1EH, UK; k.vezyroglou@ucl.ac.uk (A.V.); h.cross@ucl.ac.uk (J.H.C.); 2 Medical Physics and Biomedical Engineering, University College London, London WC1E 6BT, UK; phebden@gmail.com (P.H.); isabel.roever.13@alumni.ucl.ac.uk (I.D.R.); 3 Department of Clinical Neurophysiology, Great Ormond Street Hospital for Children NHS Foundation Trust, London WC1N 3JH, UK; rachel.thornton@addenbrookes.nhs.uk (R.T.); alan.worley@gosh.nhs.uk (A.W.); mariana.alves@gosh.nhs.uk (M.A.); emma.dean22@nhs.net (E.D.); 4 Neonatology, EGA Institute for Women’s Health, University College London, London W1T 7NF, UK; subhabrata.mitra.13@ucl.ac.uk

**Keywords:** broadband Near Infrared Spectroscopy (bNIRS), paediatric epilepsy, brain metabolism, brain injury, seizures

## Abstract

Epileptic seizures are transiently occurring symptoms due to abnormal excessive or synchronous neuronal activity in the brain. Previous functional near-infrared spectroscopy (fNIRS) studies during seizures have focused in only monitoring the brain oxygenation and haemodynamic changes. However, few tools are available to measure actual cellular metabolism during seizures, especially at the bedside. Here we use an in-house developed multichannel broadband NIRS (or bNIRS) system, that, alongside the changes in oxy-, deoxy- haemoglobin concentration (HbO_2_, HHb), also quantifies the changes in oxidised cytochrome-c-oxidase Δ(oxCCO), a marker of cellular oxygen metabolism, simultaneously over 16 different brain locations. We used bNIRS to measure metabolic activity alongside brain tissue haemodynamics/oxygenation during 17 epileptic seizures at the bedside of a 3-year-old girl with seizures due to an extensive malformation of cortical development in the left posterior quadrant. Simultaneously Video-EEG data was recorded from 12 channels. Whilst we did observe the expected increase in brain tissue oxygenation (HbD) during seizures, it was almost diminished in the area of the focal cortical dysplasia. Furthermore, in the area of seizure origination (epileptic focus) ΔoxCCO decreased significantly at the time of seizure generalization when compared to the mean change in all other channels. We hypothesize that this indicates an incapacity to sustain and increase brain tissue metabolism during seizures in the region of the epileptic focus.

## 1. Introduction

The epilepsies are a heterogeneous group of disorders sharing the predisposition to epileptic seizures as their common characteristic [1]. Phenotypes vary from, self-limiting epilepsy syndromes to difficult to treat epilepsies [2], that pose a therapeutic challenge, often necessitating the use of multiple antiepileptic drugs and sometimes alternative treatment options, such as the ketogenic diet or epilepsy surgery. Patients can experience complex comorbidities, including cognitive impairment [3]. Electroencephalography (EEG) is our gold standard for the diagnosis of epileptic seizures, and, in most cases, it can reliably detect the onset and spread of seizures [4,5]. However, it tells us little about the way seizures actually affect the brain. Investigations that offer deeper insight into brain function, such as Positron Emission Tomography (PET) [4] or functional Magnetic Resonance Imaging (fMRI) [5] are difficult to obtain during epileptic seizures.

Functional Near-infrared spectroscopy (fNIRS) is an optical technique extensively used in neuroimaging infants and children [6], providing non-invasive measurements of the changes in brain tissue oxygenation through quantification of the changes in oxygenated (HbO_2_) and deoxygenated haemoglobin (HHb). The sum of the concentration variations in HbO_2_ HHb represents the concentration change in total haemoglobin (HbT), which, under the assumption of a constant haematocrit can be regarded as a measure of regional cerebral blood volume (rCBV); while the subtraction between HbO_2_ and HHb (HbD) gives an indication of the changes in oxygenation and the mismatch between oxygenated and deoxygenated blood [6,7]. fNIRS has been used to monitor and image the brain oxygenation and haemodynamic changes during different seizure types in childhood. Generalized spike-and-wave discharges (GSWD) have been associated with an initial oxygenation in the frontal area (beginning 10 s before the GSWD) followed by strong deoxygenation [8]. More recently, diffuse optical tomography images during seizures were presented in an infant showing haemodynamic changes across the whole head [9]. A biphasic haemodynamic response was identified with increases in [HbO_2_], [HHb] and [HbT] prior to seizure onset before a decrease below baseline. Spatial variation in the haemodynamic response to seizures was observed despite generalised EEG activity. Bourel-Ponchel et al. used EEG-fNIRS monitoring to investigate haemodynamics during infantile spasms and also showed an initial increase in CBV coinciding with each spasm, followed by neurovascular coupling in all children except one patient with a large porencephalic cyst [10].

A technological extension of fNIRS is broadband-NIRS (or bNIRS) that instead of only using a pair of near-infrared wavelengths (NIR), uses 100 different wavelengths. This allows bNIRS systems to quantify not only the oxygenation and haemodynamic changes, but also the changes in oxidation status of mitochondrial enzyme cytochrome c oxidase (Δ[oxCCO]) [11]. Being the terminal electron acceptor of the electron transport chain in the mitochondria, cytochrome c oxidase (or CCO) plays a crucial role in mitochondrial oxidative metabolism, being responsible for more than 90% of ATP synthesis. Therefore Δ[oxCCO] can be used as a biomarker for cellular oxygen metabolism. In a preclinical model of hypoxic ischemic encephalopathy (HIE), Δ[oxCCO] strongly co-related with the changes in the high energy phosphates in phosphorus magnetic resonance spectroscopy (31P MRS) and recovery of Δ[oxCCO] was predictive of outcome [12]. The correlation has been confirmed in human infants with HIE in the neonatal intensive care setting [13]. Furthermore, in one neonate with HIE a progressive fall in the Δ[oxCCO] baseline was observed during repeated seizures indicating a decrease in mitochondrial oxidative metabolic state, potentially explaining the exacerbation of brain injury after repeated or prolonged seizures [14]. For further information regarding the measurement of [oxCCO] with bNIRS we recommend the reader to look at the recent review publication by Bale and colleagues [11].

Like EEG, bNIRS is a portable non-invasive technology and can be utilized at the patient’s bedside. Both of these techniques have similar temporal resolution and poor spatial resolution. Their combination however improves spatial resolution and presents a unique opportunity to investigate neurovascular and neurometabolic coupling during epileptic seizures. With this goal we developed a multimodal monitoring platform for synchronous measurements of multichannel bNIRS and EEG at the bedside of children in-between and during seizures. Here we describe our multichannel bNIRS instrument and demonstrate how the combination of EEG and bNIRS measurements of brain electrical activity, oxygenation, haemodynamics and metabolism allow a better understanding of the mechanisms and effects of epileptic events on the brain tissue.

## 2. Results

**Figure 1:**
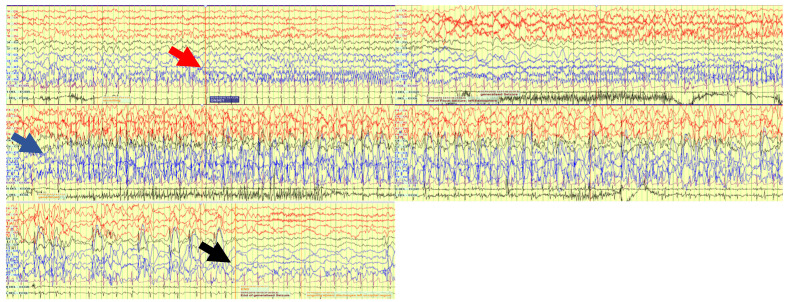
EEG trace during one of the 17 seizures. Red arrow: Seizure starts with fast activity over left posterior quadrant. Blue arrow: Seizure evolves to a generalized seizure involving all channels. Black arrow: seizure ends.

Simultaneous EEG-bNIRS was recorded continuously over 66 min. During this time 17 seizures were captured on EEG. All seizures showed a similar semiological and electrophysiological pattern. On EEG there was initial fast activity over the left posterior quadrant (P7), which evolved to spikes quickly spreading to the adjacent areas of the left central (C3, C5) and right posterior (P8) regions (see Figure 1). The seizure then generalized with spike wave activity over both hemispheres. The time of seizure onset was set at the beginning of the fast activity over P7. Median seizure duration was 59.27 s, IQR 69.77 s (min 36.33–max 132.1) seconds, and median time to generalization was 21.3 s, IQR 1.98 s (min 9.46–max 25.41) seconds. Continuous epileptiform activity (irregular slow spike waves) was seen localised over the left parietal area (P7) during the whole recording. Clinically the seizure became apparent after generalization on EEG. The patient showed blinking and twitching of the left > right side of her body.

**Figure 2:**
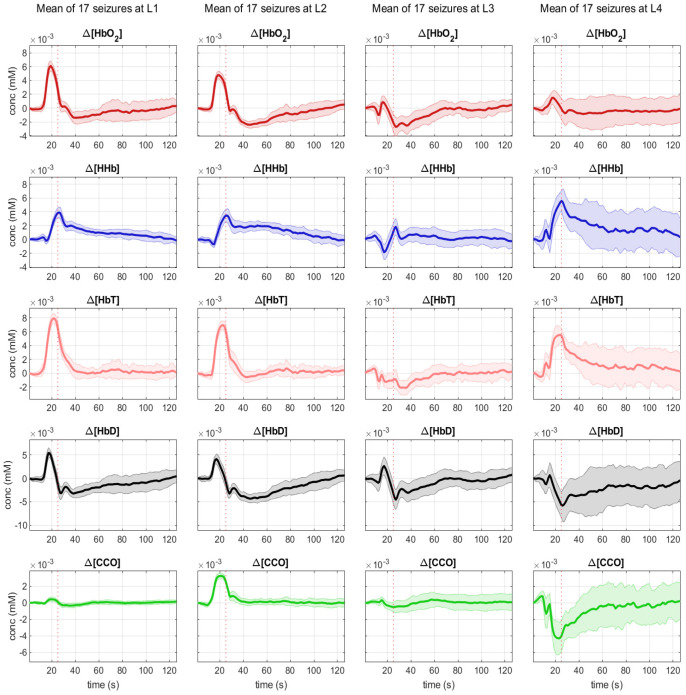

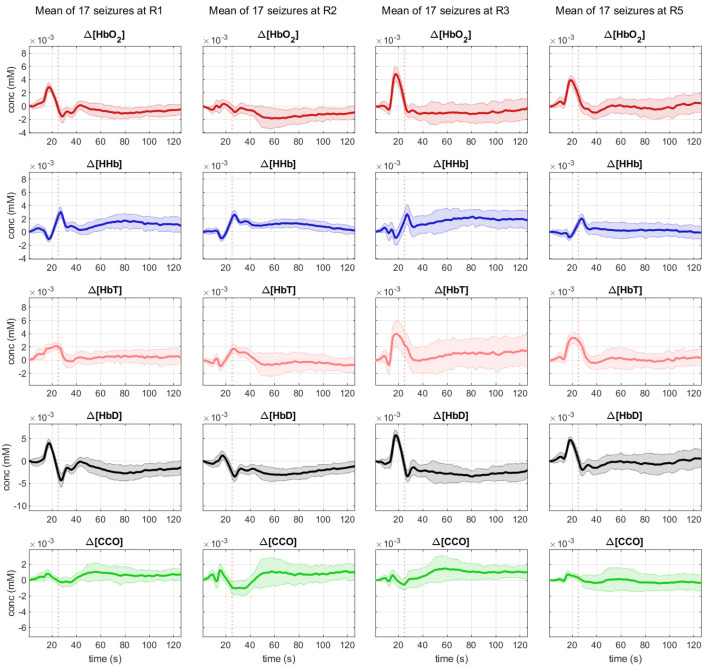
Mean changes during 17 seizures in bNIRS parameters HbO_2_ (red), HHb (blue), HbT (pink), Hbdiff (black) and [oxCCO] (green) from 20 s pior to seizure generalization (dotted line) to 100 s after seizure generalization.

We were able to record bNIRS data from 8 locations, 4 over the right (R1, R2, R3, R5) and 4 over the left hemisphere (L1, L2, L3, L4). As the patient was not able to sit up for the measurement, data from optodes recording occipitally were contaminated with artefacts. As time to seizure generalization was more consistent across the 17 seizures, than whole seizure duration, we used the time of seizure generalization (from the EEG) to align all seizures and plotted the bNIRS parameters HbO_2_, HHb, HbT, HbD and [oxCCO] over time (Figure 2). Changes in the bNIRS measurements were seen early in the seizure with major changes occurring up to the time of seizure generalization (red dotted line), before any clinical signs of the seizure had occurred. Often parameters had returned to baseline at the end of epileptic events. More specifically HbO_2_ increased very early in the seizure over all channels except R2. However, at the time of generalization this increase remained significant only over L2 and had reversed and significantly decreased over L3. After an initial decrease HHb increased across all channels and the peak of the increase coincided with time of generalization and was statistically significant over all channels except L3 and R3. The highest increase was over L4 (5.74 µmol). HbT showed the highest increase over L1, L2 and L4, moderate increase over the right hemisphere and a decrease over L3. HbD decreased across all channels with the highest decrease again over L4 (5.75 µmol/L). Finally [oxCCO] increased over L2 and decreased over L4, R1 and R2, with the most significant change at L4 (3.38 µmol/L).

**Figure 3:**
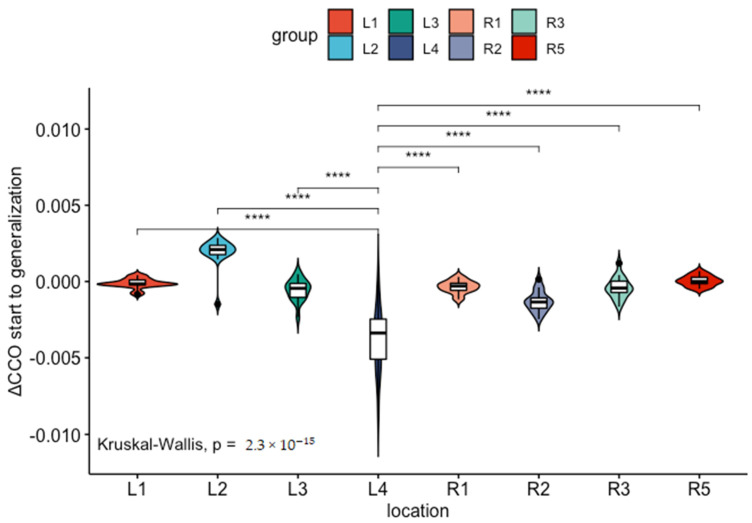
The drop in (Δ[oxCCO]) (measured in mmol) from seizure start to seizure generalization is significantly higher (****) at location L4 compared to all other channels.

Comparing these early changes across channels, we can see that position L4 has a unique profile with a significant increase in HbT comparable to L1 and L2, a significant drop in HbD comparable to L3, and a steep drop in [oxCCO] significantly greater than at any other channel measured (Figure 3).

**Figure 4:**
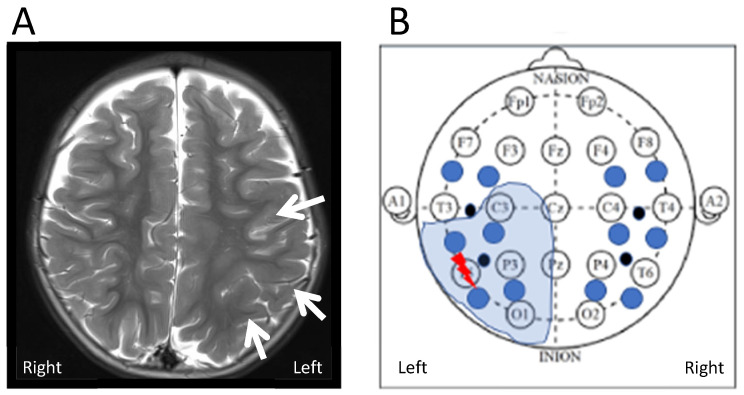
(**A**) Axial T2 weighted images on a 3 Tesla MR scanner show extensive but subtle malformation of cortical development in the left posterior temporo-parietal lobe (arrows). The malformation extends into the occipital lobe (not shown here). (**B**) Schematic correlation of region of FCD identified on MRI with EEG and bNIRS recording setup. The thunder shows the region of origin of the epileptic seizures (epileptic focus) within the extensive FCD (electrode T5). The closest bNIRS channel is L4.

The patient’s MRI brain showed an extensive focal cortical dysplasia (FCD) in the left posterior temporal, parietal and occipital lobe. It extended up to but not beyond the anomalous left central sulcus. Figure 4 shows a schematic map correlating bNIRS and EEG locations with the patient MRI. Locations L3 and L4 are collecting data from the region of cortical malformation. L4 is recording closest to EEG channel P7 where seizures originate (epileptic focus).

## 3. Discussion

Using a neuromonitoring platform for synchronous measurement of bNIRS and EEG during epileptic seizures, we have demonstrated the feasibility of this technology in the peri-ictal period in a 3-year-old child, with most significant physiological changes in the left posterior temoro-parietal region indicating the possible origin of the epileptic focus. Our unique multimodal set-up allowed us to investigate oxygenation, haemodynamics and mitochondrial oxidative metabolism alongside EEG, giving us extended insight into the origin and impact of the seizures with possible mechanism of further injury resulting from seizures. The site where seizures were shown to originate within the FCD seen on MRI had a unique metabolic signature on bNIRS, indicating a relative brain tissue hypoxia and energetic deficiency (hypometabolic state) in the region. This finding supports our hypothesis, that the combination of measurements from EEG and bNIRS can lead to better identification of epileptic foci at the patient bedside.

FNIRS has been used in the past to elucidate haemodynamics during epileptic events. Different groups have focused on different questions. So, Watanabe et al. evaluated the efficacy of fNIRS in localizing the epileptic focus with a view to use it in presurgical evaluation for epilepsy surgery [15]. Their very promising results however have not been replicated by other studies [16,17]. Other groups have focused on the use of fNIRS to identify preictal haemodynamic change, to potentially be used as a seizure warning system, with mixed results [18,19]. Nevertheless, these studies, together with others, have accumulated a wealth of information about the changes in oxygenation and haemodynamics during epileptic events, that in themselves are important to better understand seizure pathophysiology. Even though results seem to differ between studies, it seems that this is mostly due to differences in study methodology and patient groups included. Many studies only use one or two fNIRS channels placed on the patient’s forehead [14,18,19]. Depending on the type of seizure measured and its origin within the cortex, results are difficult to compare. However, focusing on multichannel studies [15,16,17,20,21,22,23] and the haemodynamic response measured close to the epileptic focus, the picture is more consistent. With the exception of a study of posterior seizures [23], all authors describe an increase of HbO_2_ and rCBV during epileptic events. Some authors describe an initial dip in HbO_2_ [21] or a slight delay (<2 s) in its increase [17], but overall the observation is coherent with the ictal hyperperfusion also described with alternative methods, such as single-photon emission computerized tomography (SPECT). In our study we also see ictal hyperperfusion near the focus (L1, L2), however HbO_2_ increase is limited in the area of cortical malformation including the epileptic focus (L3, L4). The HHb response is more varied, often initially decreasing, but then increasing as the seizure progresses. This is coherent with our observations, and is possible evidence, that the attempt to meet increased metabolic demands during seizures through increased perfusion, ultimately fails, leading to a metabolic mismatch, that sometimes exceeds the duration of the seizure.

Malformations of cortical development (MCDs) occur when normal processes of cortical development are disrupted during fetal development. Depending on when the disruption occurs, normal cortical neurons may be mispositioned or abnormal cortical neurons present. The resulting imbalances between excitatory and inhibitory systems are believed to drive the epileptic seizures that often accompany MCDs. It is estimated that 25–40% of drug-resistant epilepsy in childhood is caused by MCDs, even though their exact incidence is unknown [24]. Focal cortical dysplasias (FCDs) are localized MCDs, formerly believed to mostly result from a toxic insult to the developing brain, but recently also associated with DEPDC5 mutations [25] and somatic mutations in MTOR, PIK3CA, AKT3, PTEN, TSC1 and TSC2 [26]. Their size can vary greatly, and their anatomic location will define the semiology of resulting epileptic seizures. MRI is the mainstay for FCD diagnosis; even though MRI presentation can be subtle. In these cases fluorodeoxyglucose-positron emission tomography (FDG-PET) can be a helpful additional imaging modality, as focal cortical dysplasias typically show focal regional hypometabolism on FDG-PET imaging even in MRI-negative cases [25].

Our patient has a large FCD of the left posterior temporal, parietal and occipital lobes with continuous epileptic activity and frequent seizures originating in the left parietal lobe. All seizures originate from the same region within the FCD, the epileptic focus, located near EEG channel P7 and bNIRS channel L4. It is here that we measure the most significant changes in haemodynamics and neurometabolism. HbO_2_ fails to increase, as commonly described in epileptic events. Instead, there is a significant increase in HHb and a significant drop in oxCCO. At channel L3, the other channel within the dysplastic area, but outside the epileptic focus, HbO_2_ still fails to increase, but there is no equivalent drop in oxCCO. Moving outside the dysplastic area to channels L1 and L2 we see a haemodynamic signature more in line with the ictal hyperperfusion described by other groups with an increase in HbO_2_, followed by a smaller increase in HHb. The failure of HbO_2_ to increase within the FCD during seizures might be accentuated by the dysplastic tissue, however the significant decrease in oxCCO is unique to the area of the epileptic focus with near constant epileptic activity and it seems likely that the seizures are driving a relative brain tissue hypoxia and energetic deficiency in the region. Our group has previously described seizure induced hypometabolism in neonates with hypoxic ischaemic encephalopathy [14]. Hypometabolism has also been shown in patients with drug resistant temporal lobe epilepsy and animal models after induced status epilepticus [27]. So, we hypothesize that, even though during most seizures neurovascular coupling remains intact to meet the increased demand for oxygen and glucose consumption, in some clinical scenarios of underlying damaged brain tissue (FCD, HIE, posttraumatic brain injury), very frequent seizures or prolonged status epilepticus this mechanism can break down, leading to a seizure induced hypometabolic state, potentiating neuronal injury and negatively impacting outcome. Broadband NIRS allows us to identify this hypometabolic state in real time at the patient’s bedside.

We are aware, that our study is limited by the fact, that it is a one patient case study. However, the fact that our haemodynamic and neurometabolic measurements are highly consistent throughout 17 separate epileptic seizures makes us confident, that the measurements will be reproducible in other individuals. In addition, further work is needed to improve the attachement of the bNIRS probes on the head when applied together with EEG to ensure good quality data with less motion artifacts. Possible solutions can involve a cap that better integrates the EEG and bNIRS probes.

The combination of EEG with bNIRS, including oxCCO measurement, allowed us a unique insight into oxygenation and cell metabolism during epileptic events in our patient through a non-invasive measurement done at the patient’s bedside. In our patient the combination of different measurements revealed separate metabolic profiles for areas of healthy tissue (L1, L2) with HbO_2_ increase and stable oxCCO, dysplastic areas (L3, L4) with failure of HbO_2_ increase, as well as the actual area of the epileptic focus (L4) with failure of HbO_2_ increase and significant oxCCO drop. This could develop into a useful clinical tool aiding the identification of the epileptic focus, as well as the diagnosis of a cortical malformation in patients with drug-resistant epilepsies and lesion negative MRI. Broader studies are needed to compare these results to further patients with both, cortical malformations and focal epilepsies of different origin.

## 4. Materials and Methods

### 4.1. Patient Recruitment

We are reporting an interesting case study of a 3-year-old girl with an extensive left posterior focal cortical dysplasia (FCD) in Great Ormond Street Hospital in London, UK. We obtained informed parental consent to perform a bNIRS-measurement alongside her planned EEG recording. The study was approved by the local Research Ethics Committee (REC reference: 17/LO/1402).

### 4.2. EEG Instrument

The EEG was performed by an EEG technician experienced in paediatric EEG, as part of the patient’s clinical care. A reduced set of electrodes was used, as the EEG had to be performed at the patient’s bedside. The locations recorded were A1, A2, F3, F4, Fpz, C3, C4, Cz, T3, T4, T5, T6 (10-10 system). The EEG trace was analyzed by an experienced paediatric neurophysiologist to determine the timings of the beginning, generalization and end of the seizure episodes.

### 4.3. MRI

We reviewed the MRI images of our patient. These had been taken 4 months prior to the simultaneous EEG-bNIRS measurement, as part of the clinical care of the patient. MRI was performed on a 3 Tesla MRI scanner (Siemens, Munich, Germany) following the Great Ormond Street Hospital epilepsy neuroimaging protocol. This study sequences included 3D T1-MPRAGE, T2-FLAIR 1mm with reformants, axial and coronal T2 axial DWI, axial DTI and axial SWI.

### 4.4. Multichannel bNIRS Equipment

**Figure 5:**
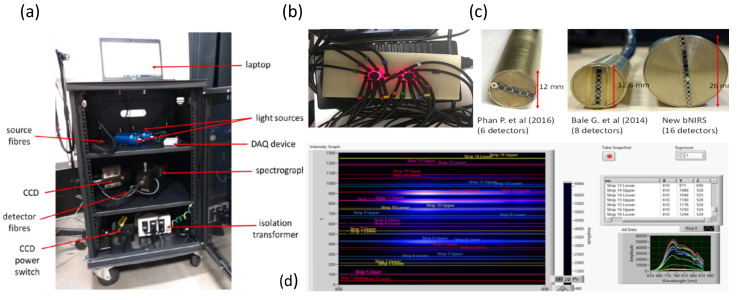
(**a**) Picture of the 16 detector bNIRS system (only 14 detectors used); (**b**) Picture of source and detector fibres on a solid phantom; (**c**) Detector ferrule comparison from previous instruments Phan P. et al. (2016) (16), Bale G. et al. (2014) (14) and current instrument; (**d**) Image of the software acquisition illustrating the CCD binning for each detector.

The multichannel bNIRS imaging system is an in-house developed instrument that is composed of two broadband tungsten halogen light sources, (HL-2000, Ocean Optics), shown in Figure 5a, that emit light between 360–2400 nm. A 650 nm long-pass filter is used to remove shorter wavelengths and hence minimize heat deposition and UV exposure applied to tissue. Light source fibres custom-built (Loptek, Germany) were used to transmit the light to the head. The bundle of fibres had a high numerical aperture with a bundle diameter of 2.8 mm surrounded by an outer casing of 10 mm. Fibres were 2 m long and connected to the light sources via an SMA connector. Reflected light from the head collected by customized fibres (Loptek, Germany) with a numerical aperture of 0.57; this allowed for a minimum detector head height of 5 mm with fibres still being able to bend 90 degrees from the head surface. Each detector fibre consists of a fibre bundle with diameter of 1 mm and a casing of 5 mm. The sixteen detector fibres are bundled vertically into a ferrule with a 25 mm diameter, with each fibre bundle separated by 0.5 mm casing (see Figure 5b–d). The Acton Series LS785 spectrometer (Princeton Instruments, Trenton, NJ, USA) was used to separate the incoming light into its wavelength components. It is a lens-based spectrograph enabling a higher throughput than mirror-based systems with fast f/2 lenses enabling direct fibre optic coupling. Anti-reflective coated lenses provide optimum NIR transmission (between 750–1050 nm) of over 99% transmission throughout the working range of the spectrograph. The diffraction grating inside the spectrometer has a bandwidth of 308 nm, with a wavelength range between 610 nm and 918 nm. The grating is blazed at 750 nm and has 600 grooves/mm. After light has been diffracted at the grating, it is focused (with an f/2 lens) onto a charge-coupled device (CCD) the PIXIS 1300F (Princeton Instruments, USA). It is a front-illuminated CCD with a two-dimensional imaging array, with a sensor size of 26.8 × 26 mm. This sensor contains a 1340 × 1300 pixels imaging array, with pixel size is 20 × 20µm.

This newly developed multichannel bNIRS system represents a significant upgrade to previous instruments by Bale et al. [13] and Phan et al. [28]. It has the capacity to measure changes in light attenuation of 308 near-infrared (NIR) wavelengths (610 nm to 918 nm) and using the University College London (UCL)n algorithm quantify the changes in oxy-, deoxy-haemoglobin concentration (HbO_2_, HHb) and oxCCO simultaneously over 16 different brain locations with a sampling rate of 2 Hz. A picture of the system is shown in Figure 5a.

### 4.5. Optode Placement

**Figure 6:**
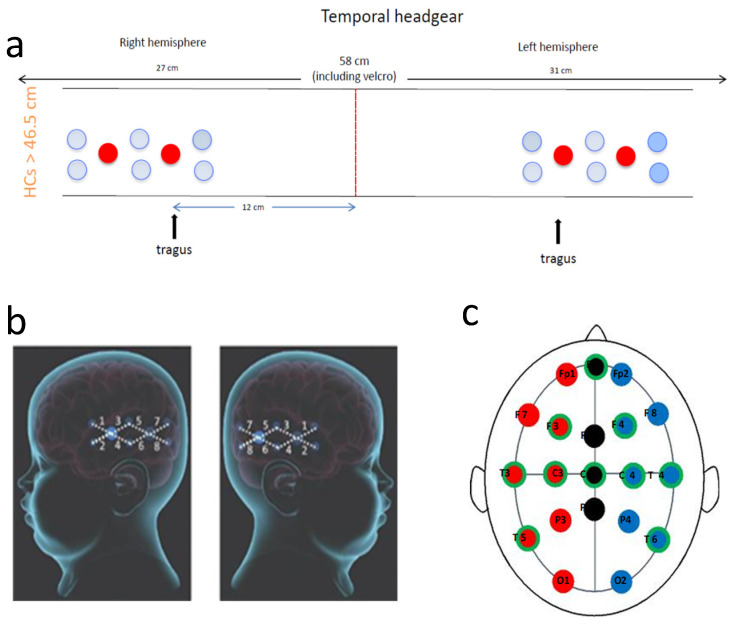
Recording setup. (**a**): Schematic presentation of the bNIRS headband used. Eight channels were placed over each temporal lobe. The red line shows centre front. The front light source was placed over the tragus on both sides; (**b**): Diagram showing bNIRS channel locations across the left and right temporal lobes (large blue circles are the sources and the smaller one are the detectors with 25 mm source-detector distance); and (**c**): Diagram showing EEG electrode locations with the electrode positions used in this study circled in green.

Due to the young age of the patient and the fact that the seizures originated focally from the left posterior hemisphere, the bNIRS headband was placed to monitor both left and right hemispheres over the temporal regions (8-channels on the left and 8-channels on the right) (see Figure 6). The bNIRS and EEG were put on simultaneously; this enabled careful placement of the EEG electrodes around the bNIRS probes. The placement of the NIRS probes to maximise signal quality was especially important using the temporal band due to the added complication of hair, which can significantly attenuate the bNIRS signal. The bNIRS probe array was initially held against the side of the head as a guide and EEG electrodes were glued to the head in a symmetric configuration according to the 10–20 EEG system. The EEG electrode positions are shown in Figure 6 where the electrodes used in this study are shown circled in green.

The bNIRS headband was placed on after the EEG such that the front light source was located above the tragus on both sides. A source-detector separation of 2.5 cm was used. Monitoring was performed for an hour with no intervention. Figure 2 shows the location of the bNIRS array overlaid on an image of a toddler’s brain.

The sampling rate of the bNIRS system is 2 Hz. A trigger was pushed at the start of the bNIRS measurement to mark the exact time on the ongoing EEG recording for offline synchronisation.

### 4.6. Data Pre-Processing

The quality of the bNIRS data was initially assessed before further analysis was performed, including assessment of the raw intensity spectra (check if intensity spectra for each detector was above 600 counts, which was the dark noise count) and the implementation of residual analysis to check the validity of using the three-chromophore fit model to derive concentration changes [11]. Eight channels were discarded from further analysis due to artefact contamination, 4 channels from the left and four channels from the right hemisphere. Changes in concentration of HbO_2_, HHb and oxCCO were calculated using the UCLn algorithm between 770–906 nm, with the wavelength dependency of the DPF applied. A DPF of 5.15 was used based on the calculation from Duncan et al. [29] at 807 nm where the age of the subject is taken into account. bNIRS data was finally resampled to 1 Hz using spline interpolation.

EEG data was sampled at 256 Hz and filtered to reduce low frequency noise such as movement artifacts and high frequency components such as electromyography signals. A filter constructed from a second order Butterworth filter and a zero phase filter was used and applied in cascade to promote filter stability. First a 35 Hz low-pass filter was used followed by a 0.5 Hz high-pass filter. Use of a zero phase filter helped preserve features without distorting the temporal components of the signal.

### 4.7. Analysis

R studio was used to perform statistical analysis. For every channel we calculated the amplitude change in the 5 parameters HbO_2_, HHb, HbT, HbD andoxCCO over 3 time periods: 20 s before seizure onset to seizure onset (baseline), seizure start to seizure generalization and seizure start to seizure end (whole seizure); we did this by substracting the value at the end of the time period from the value at the beginning. The time periods were identified from the EEG trace and events. We then compared the differences between the three time periods, using non-parametric tests (Wilcoxon and Kruskal-Wallis Test). The same tests were used to compare the change in parameters between the different brain locations (channels) we were measuring at. A cut off for *p*-value 0.5 was used for significance.

### 4.8. Case Report

We describe a 3-year-old girl, who first presented with seizures at the age of four months. Seizures were described as episodes of hypotonia, eye rolling and subsequent stiffening of her right side. Seizure durations were around 15 min and seizures occurred every 2–3 weeks. Antiepileptic treatment was initiated with phenobarbitone and seizure semiology evolved to clusters of hypotonic episodes with fluttering of the right eye. Seizure freedom was not achieved. Over the next 2 years several anti-epileptic medications (AEDs) including Levetiracetam, Lamotrigine, Clonazepam, Phenytoin, Vigabatrin and three courses of steroids were used without success. When we first met her at age 2.5 years, she was on triple therapy with Sodium Valproate, Topiramate and Clobazam. Her seizures exacerbated and she was transferred for urgent presurgical evaluation for epilepsy surgery.

A video-EEG data recorded from 12 channels (A1, A2, F3, F4, Fpz, C3, C4, Cz, T3, T4, P7, P8). revealed continuous epileptiform sharpened activity over the left posterior quadrant. During clinical events the EEG showed fast activity over the left parietal region evolving to widespread sharp and slow waves over the left hemisphere and then evolving over both hemispheres.

## 5. Conclusions

Identification of the epileptic focus in an epileptic patient is important for both, accurate diagnosis and understanding of the epilepsy network and exploration of therapeutic options, foremost epilepsy surgery. We were able to quantify unique changes in brain tissue oxygenation and cellular metabolism at the epileptic focus during epileptic seizures in a paediatric patient. Changes occurred early in the events before clinical manifestations of the seizures were visible. HbO_2_ failed to increase in the region of cortical malformation and Δ[oxCCO] significantly decreased only in the region of the epileptic focus, possibly indicating an incapacity to sustain and increase brain tissue metabolism during seizures and resulting in a hypoxic and hypometabolic state. The combined monitoring of bNIRS and EEG offered unique insights into the location of the epileptic focus, as well as the changes in brain physiology (oxygenation, haemodynamics and mitochondrial metabolism) during epileptic events.

## Data Availability

The data presented in this study are available on request from the corresponding author. The data are not publicly available due to patient related data.
